# Prevalence and factors associated with *Helicobacter Pylori* infection among children with sickle cell anemia attending Mulago hospital, in Uganda

**DOI:** 10.4314/ahs.v22i2.16

**Published:** 2022-06

**Authors:** Idris Swaleh Mubiru, Phillip G Kasirye, Heather Hume, Grace Ndeezi

**Affiliations:** 1 Department of Paediatrics and Child Health, School of Medicine, Makerere University College of Health Sciences; 2 Department of Paediatrics, Mulago National Referral Hospital; 3 Dept of Paediatrics, Universite de Montreal, Canada

**Keywords:** Recurrent abdominal pain, Sickle cell anemia, Dyspeptic symptoms

## Abstract

**Background:**

Children with sickle cell anemia (SCA) have a high predisposition to a range of infections and gastrointestinal disorders. Studies of children living in low income countries have shown high levels of infection with *Helicobacter Pylori* (*H. pylori*), however, there are no reports in Ugandan children with SCA.

**Objectives:**

We aimed to describe the prevalence and factors associated with *H. pylori* infection among children with SCA at Mulago Hospital.

**Methods:**

A cross-sectional study was conducted on 340 children with SCA aged 5–18 years. Assessments included recurrent abdominal pain(RAP), dyspeptic symptoms, relevant medical and social histories. Stool samples were collected and an antigen test carried out to determine *H. pylori infection. H. pylori* prevalence and its associated factors were determined.

**Results:**

*Helicobacter pylori* infection was detected in 49%(168/340); (95%Confidence interval (CI): 44.1, 54.7) of the study subjects. Having epigastric pain was independently associated with *H. pylori* infection; (Adjusted odds ratio [aOR] = 1.89; 95%CI: 1.1, 3.6; p= 0.048). Pneumococcal vaccination; (aOR=0.425; 95%CI: 0.2, 0.9; p=0.019) and appetite loss; (aOR=0.588; 95%CI: 0.3, 0.9; p=0.046) were negatively associated with *H. pylori* infection. RAP was not associated with *H. pylori* infection.

**Conclusions:**

*H. pylori* infection was common among children with SCA and independently associated with epigastric pain but not recurrent abdominal pain. Pneumococcal vaccination and appetite loss were protective against the infection. Screening for *H. pylori* should be carried out in SCA children with epigastric pain.

## Introduction

Sickle cell disease is the commonest genetic disorder worldwide with the highest frequencies of homozygous (HbSS) occurring in sub-Saharan Africa, where 3– 4% of the population are affected^1^. According to the World Health Organization (WHO), 5% of the world population carries the sickle cell gene which is responsible for sickle cell anemia (SCA) and thalassemia^2^. Furthermore an estimated 300,000 infants are born globally each year, two thirds of these occurring in Africa where the disease has a 5% contribution to the under-five mortality^2^. The genetic defect arises from a substitution of glutamic acid by valine in position 6 of the beta-globin chain resulting in the formation of an abnormal hemoglobin molecule (HbS), which is responsible for the disease presentations. Children with sickle cell anemia have a high predisposition to infections by encapsulated bacterial infections, such as Streptococcus pneumoniae, Haemophilus influenzae, salmonella species, viral infections like Parvovirus B19, among others^3^. This is in part due to the recurrent blood transfusions they get as well as the complication of functional asplenia resulting from infarctions. *Helicobacter pylori* (*H. pylori*) infection, a gram negative, micro aerophilic spiral, bacterial organism associated with peptic ulcer disease, chronic active gastritis and recurrent abdominal pain may also be as or more common than in the general population^3^,^4^. It is acquired early in life and dwells in the mucous layer of the gastric mucosa of the human stomach overlying the epithelium^5^.

Recurrent abdominal pain, a common presentation in sickle cell anemia could be due to several factors including; *H. pylori* infection, cholecystitis, ischemic bowel injury, paralytic ileus, splenic sequestration, acute hepatic syndromes and or gastritis related to prolonged use of non-steroidal anti-inflammatory drugs 6–9.

Global rates of *H. pylori* infection vary from one region to another but estimates suggest that close to half of the world population is infected with this organism, with higher rates reported in developing countries^10^, ^11^. Many of the infected individuals are asymptomatic but can present with chronic gastritis associated with recurrent peptic ulcers and cancer 12, 13.

Chronic hypoxia/ infarction, anemia, and the increased use of non-steroidal anti-inflammatory drugs (NSAIDs) for pain management are culpable further promoting the growth and survival of *H. pylori* in children with SCA^4^. Some studies have reported *H. pylori* infection as an independent risk factor for peptic ulceration among chronic NSAID users. Equally anemia has been attributed to peptic ulceration in non NSAID users^14^. This then indirectly implicates *H. pylori* in the causation of gastritis and gastric ulcer pains 15, 16. Both of these could explain recurrent abdominal pain in children with sickle cell anemia.

The burden of bacterial infections has been studied among Ugandan children with sickle cell anemia^17^. The prevalence of *H. pylori* infection among sick children presenting to Mulago Hospital was found to be 64% in an earlier generalstudy(18). However, this was not limited to children with sickle cell anemia who may have more abdominal complaints.

We set out to establish the prevalence and factors associated with *H. pylori* infection among children with SCA at the largest care centre in Uganda given that its effects could mimic an abdominal crisis where appropriate treatment could be missed.

## Methods

### Study design and setting

We employed a cross-sectional study design to collect data at the Mulago Hospital sickle cell clinic, Kampala Uganda between February and May 2016. This is the oldest and largest care center for sickle cell anemia in Uganda. The clinic runs from Monday to Friday (8:00am to 4:00pm) and attends to an average of 30–40 patients each day.

### Study population

This included children aged 5 to 18 years with confirmed sickle cell anemia by hemoglobin electrophoresis. Consent was obtained from the caregivers and assent from children above eight years. We excluded children who had used any combinations of medicines used in *H. pylori* eradication therapy which included omeprazole, metronidazole, amoxicillin and clarithromycin or any proton pump inhibitor within two weeks of the clinic visit^19^. We also excluded any child with cognitive impairments secondary to stroke or any other cause because of their inability to describe abdominal pain.

### Sample size and sampling procedure

The sample size of 340 children for the prevalence of *H. pylori* was calculated using Kish Lislie formula (1965) with a 95% confidence level, 5% degree of precision and a prevalence of *H. pylori* infection among children with sickle cell disease of 67.8% from a Nigerian study^4^. Children who fulfilled the inclusion criteria were consecutively enrolled into the study. Up to 20 children were sampled daily, selected from five different age brackets by the research assistants and or Principal Investigator.

### Data collection method and procedures

Prospective participants were identified as they presented for routine care at the clinic and informed consent sought before inclusion. Study aims, procedures, benefits and potential risks or discomfort were explained to the study participants and their caretakers as part of the consenting process. A care taker was considered to be the person who attends to and cares for a child while at the sickle cell clinic. Research assistants included two nurses and one medical officer all trained prior to study commencement.

The caretakers of children provided informed consent on behalf of children aged five to seven years. They were provided with consent forms to read through and given opportunity to ask questions for which the investigator and or research assistants explained anything that was not clear. For children aged eight to eighteen years, assent forms were given and depending on their ability to read, it was read to or with them. The children were given an opportunity to ask questions and the investigator or research assistant took opportunity to explain anything not clear. Thereafter, parental permission was sought from the caretaker to allow the child to participate in the study.

A standardized pretested and pre-coded interviewer administered questionnaire was then administered where consent was obtained and key information included; history of recurrent abdominal pain or presence of dyspeptic symptoms (epigastric pain, nausea, generalised abdominal pain, anorexia and flatulence amongst others). Other symptoms assessed for included weight loss, fever, vomiting and diarrhea. Recurrent abdominal pain was considered when a child reported at least three episodes of severe upper abdominal pain that affected normal functioning and required medical attention, within the three months preceding the clinic visit^20^.

Current abdominal pain was considered as generalized abdominal pain at presentation. Patients were also assessed for medication use, other relevant medical and social history. For most of the children who participated in the study, it was their caretakers who were interviewed. Older children above the age of eight years were involved in some parts of the interview where the caretaker didn't have full information, especially with the description of symptoms. The children were interviewed in presence of their caretakers were they felt more comfortable and their responses were appropriate.

Clinical signs included: Temporal muscle wasting which was considered as reduced muscle bulk as determined by observation or palpation of the peripheral muscles, body weight, height, nutritional status, presence of pallor, jaundice, hepatosplenomegaly, and epigastric tenderness. A calibrated digital weighing scale was used and readings to the nearest 0.1kg recorded. Height was measured using a Kawe height measure tape mounted on the wall. Study participants were assessed with no shoes and findings recorded to the nearest 0.1cm. Two measurements were taken and if there were any inconsistencies a third measurement was taken. An average of the two closest readings was taken as the correct measurement. Body mass index (BMI) was calculated for each child and BMI percentile for age recorded. A BMI for age below the 5th percentile was considered as wasting.

### Collection of blood samples

Blood samples were drawn from patients following standard procedures. The skin over the antecubital fossa was cleaned using 70% alcohol followed by the drawing of 1–2mls ml of blood into an EDTA vacutainer for complete blood count assessment at Mulago Hospital hematology laboratory using a Hematological analyzer MEK-7222 (UNIT1) celltac E brand.

### The stool antigen test for *H. pylori*

Stool was collected either at the time of contact or later in the day for those who were unable to provide stool immediately and transported to the microbiology laboratory of Makerere University in cold boxes within 2 hours of collection at a temperature of 2 to 80C until the analysis.

*Helicobacter pylori* infection was determined by using a stool antigen test kit SD Bioline brand from the republic of Korea. The kit was 95.6% sensitive and 92.5% specific(21). For the process, 50mg of the stool sample was mixed with the assay diluents at room temperature and after complete dissolution, 3 drops of the prepared sample were added into the sample well of the test device. A positive test was considered when both the test band and the control band appeared within the result window of the test device while a negative test was considered if only the control band appeared.

### Data Management and analysis

All questionnaires were cross checked for completeness, data audited using frequency distributions and cross tabulated to detect missing and out of range/ illogical values.

The questionnaires and laboratory results were stored under lock and key to maintain confidentiality. The questionnaire was pretested, standardized and translated into the commonly spoken local language which is Luganda, before the data collection which was closely supervised by the Principal Investigator. Pretesting of the questionnaire was done at the paediatric outpatient department of Mulago National Referral Hospital from one of the paediatric specialized clinics, by the Principal Investigator. Three children aged between 5 and 18 years together with their caretakers were selected for the exercise. The respondents answered the questions the way the research was intended, questions were found to be easy to understand and the questionnaire was found to be comprehensive. Standardization of the questionaire was done by a biostatistician, who used the recent data collection tools used for studies done at the sickle cell clinic, and those done on *H. pylori* and recurrent abdominal pain in the local setting as standards.

EPI DATA version 3.1 was used for data entry and STATA 12.0 for analysis. Baseline characteristics were summarized in proportions, means and medians. Continuous variables were analyzed using means or medians and their measures of dispersion while categorical variables were summarized using frequencies and percentages. The prevalence of *H. pylori* was determined as the proportion of children with a positive stool test reported as a percentage with its confidence intervals. The factors associated with *H. pylori* infection were analyzed; at the bivariate level basing on the outcome of the *H. pylori* test. A student's t – test was used for continuous and the chi-square or Fisher's exact test for categorical data. Variables with p-values of less than 0.2 were considered for multivariate analysis where those with a p-value of less than 0.05 were considered significant. Analysis at this level was performed using the stepwise logistic regression model.

Laboratory quality was observed by; having inbuilt positive controls, double checking with prior established positive controls and storing the stool samples at temperatures of 2–80 C before the analysis. In addition, two technicians conducted the analysis for quality. The two technicians agreed on the final results from each sample, after sharing opinions on the test samples which were difficult to interpret.

### Ethical considerations

Ethical approval was obtained from the school of medicine research and ethics committee and consent obtained from the caretakers by the principal investigator/research assistant. The study was approved by the committee under the study protocol approval number, #REC REF 2015-142.

## Results

### Respondents' characteristics

A total of 370 children attending the sickle cell clinic were screened for enrolment into the study. Of these, one child did not meet the inclusion criteria because he did not have a confirmed diagnosis of sickle cell anemia. Of the 369 children who met the study inclusion criteria, 29 children were excluded because of having used omeprazole or any combination of drugs used in *H. pylori* eradication. These drugs included omeprazole or any other similar drug that falls in the class of drugs called proton pump inhibitors, metronidazole, clarithromycin and amoxicillin. Eventually, 340 children were consented and enrolled into the study.

### Demographic and baseline characteristics of the study participants

Girls constituted the majority at 187 (55%) and the mean age was 9.3 years, standard deviation SD (3.3), ranging between 5 and 18 years. Majority of the study participants came from Wakiso (44%) and Kampala (40%) districts. Ninety three percent of the caretakers were females, largely mothers. Majority (61%) of the care takers had obtained education beyond primary level and half of them were self-employed. Other demographic characteristics are shown in table 1.

**Table 1:** Baseline characteristics of the study participants

VARIABLE	FREQUENCY(N=340)	PERCENTAGE(%)
**Age of the participant child**		
<12	252	74
>=12	88	26
**Sex of the child**		
Male	153	45
Female	187	55
**District of residence**		
Kampala	137	40
Wakiso	151	44
Other	52	15
**Age of the caretaker**		
<=30	112	33
31–40	160	47
>40	68	20
**Sex of the caretaker**		
Male	24	7
Female	316	93
**Relationship of caretaker** **with child**		
Mother	293	86
Father	19	6
Other	28	8
**Occupation of caretaker**		
Professional	44	13
Self Employed	171	50
Peasant Farmer/others	30	9
Housewife	95	28
**Highest level of education of** **caretaker**		
Not more than primary	132	39
Beyond primary	208	61
**Type of residence**		
rural	82	24
urban	258	76

### Clinical findings

**Figure 1:**
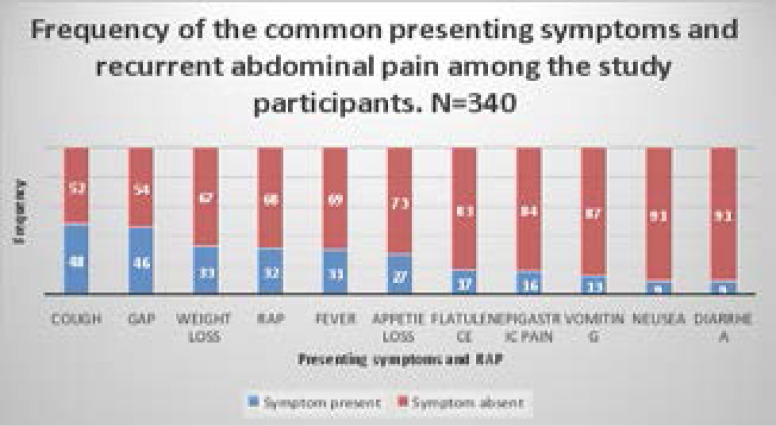
A stacked column bar graph showing the distribution of the common dyspeptic symptoms and other common presenting symptoms among the study participants. RAP= Recurrent abdominal pain, GAP; Generalized current abdominal pain

The commonest presenting symptoms of the study participants included; cough, generalized abdominal pain, weight loss, fever, loss of appetite, perception of abdominal fullness/gas, epigastric pain, vomiting, nausea and diarrhea, in reducing order of frequency. The frequency of these presenting symptoms among the study participants ranged between 9% and 48%. Recurrent abdominal pain was also found in 34% of the study participants. Details are shown in figure 1.

### Prevalence of *H. pylori* infection and associated factors

The overall prevalence of *H. pylori* infection among these children was 49% (168/340) (95% Confidence Intervals (CI): 44.1, 54.7). Out of the 168 children who tested positive for *H. pylori* infection, 74% (125/168) were below the age of 12 and 45% (76/168) were males. At bivariate level we included; social demographic characteristics, clinical characteristics and laboratory findings as variables for association with the *H. pylori* infection. Having had epigastric pain at presentation (Odds ratio (OR) 1.892; 95%CI: 1.0, 3.4; p-values 0.034) was positively associated with H. pylori infection whereas pneumococcal vaccination (OR 0.381; 95%CI: 0.2, 0.8; p-values.006) was negatively associated with *H. pylori* infection. Other factors including temporal wasting (OR 2.114; 95%CI: 0.7, 6.3; p-values 0.18) and less frequent use of painkillers like paracetamol, NSAIDs and morphine, at intervals longer than one month, (OR 1.557; 95%CI: 0.8, 3.0; p-values 0.177) were positively associated with *H. pylori* infection but the association was not statistically significant. Frequent use of painkillers was an indicator of frequency of pain episodes in these children. Of note there was no association between *H. pylori* infection and recurrent abdominal pain (OR 1.008; 95%CI: 0.6, 1.6; p-values 0.974) . At multivariate analysis, presence of epigastric pain at presentation remained significantly associated with *H. pylori* infection (Adjusted odds ratio (aOR) 1.890; 95%CI: 1.1, 3.6; p-value 0.034). Children who had epigastric pain at presentation were 1.89 times more likely to be infected with *H. pylori* when compared to those who did not have epigastric pain at presentation. Negatively associated factors with *H. pylori* infection included; pneumococcal vaccination (aOR 0.425; 95%CI: 0.2, 0.9; p-value 0.019) and appetite loss at presentation (aOR 0.588; 95%CI: 0.3, 0.9; p-value 0.046). The odds of being infected with *H. pylori* were reduced by 41% among children who had lost appetite at presentation, and 57.5% among those who had pneumococcal vaccination. Details of these analyses are summarized in table 2.

**Table 2:** Factors associated with H. pylori infection among study participants

	H.pylori		OR(95% C I)	P-value	a OR (95%CI)	P-value
	Negative	Positive				
**Age of the participant child**						
<12	127(74)	125(74)	1			
>=12	45(26)	43(26)	0.971(0.6–1.6)	0.905		
**Sex of the child**						
Male	77(45)	76(45)	1			
Female	95(55)	92(55)	0.981(0.6–1.5)	0.931		
**Main water source for the household**						
Tap	102(59)	107(64)	1			
Well	30(17)	22(13)	0.699(0.4–1.3)	0.253		
Borehole	18(10)	17(10)	0.900(0.4–1.8)	0.774		
Spring	13(8)	16(10)	1.173(0.5–2.6)	0.688		
Tank	9(5)	6(4)	0.636(0.2–1.8)	0.405		
**Toilet facility of the household**						
Water closet	19(11)	17(10)	1			
Pit latrine	153(89)	151(90)	1.103(0.6–2.2)	0.781		
**Highest level of education of caretaker**						
Not more than primary	66(38)	66(39)	1			
Beyond primary	106(62)	102(61)	0.962(0.6–1.5)	0.863		
**Recurrent abdominal pain**						
No	117(68)	114(68)	1			
Yes	55(32)	54(32)	1.008(0.6–1.6)	0.974		
**Epigastric pain**						
No	151(88)	133(79)	1		1	
Yes	21(12)	35(21)	1.892(1.0–3.4)	0.034*	1.890 (1.1–3.6)	0.048*
**Appetite loss**						
No	118(69)	131(78)	1		1	
Yes	54(31)	37(22)	0.617(0.4–1.0)	0.052	0.588(0.3–0.9)	0.046*
**Temporal wasting**						
No	167(97)	158(94)	1		1	
Yes	5(3)	10(6)	2.114(0.7–6.3)	0.18	2.542(0.8–7.9)	0.105
**Hepatomegaly**						
No	86(50)	96(57)	1		1	
Yes	86(50)	72(43)	0.75(0.5–1.1)	0.187	0.872(0.5–1.4)	0.566
**Epigastric tenderness**						
No	132(77)	139(83)	1		1	
Yes	40(23)	29(17)	0.688(0.4–1.2)	0.171	0.582(0.3–1.0)	0.071
**Pneumococcal vaccination**						
No	141(82)	155(92)	1		1	
Yes	31(18)	13(8)	0.381(0.2–0.8)	0.006*	0.425(0.2–0.9)	0.019*
**Penicillin prophylaxis**						
No	155(90)	150(89)	1			
Yes	17(10)	18(11)	1.094(0.5–2.2)	0.801		
**Use of pain killers**						
No	14(8)	14(8)	1			
Yes	158(92)	154(92)	0.975(0.4–2.1)	0.948		
**Frequency of painkiller use**						
Less than a month	27(17)	18(12)	1		1	
At intervals longer than 1 month	131(83)	136(88)	1.557(0.8–3.0)	0.177	1.529(0.8–3.0)	0.208
**Hemoglobin concentration**						
<7g/dl	74(43)	61(36)	1			
>=7g/dl	98(57)	107(64)	1.325(0.9–2.0)	0.206		

## Discussion

This study determined the prevalence of *H. pylori* among children with sickle cell anemia aged 5–18 years attending Mulago National Referral Hospital. It also sought to establish the factors that are associated with this infection in the same population. *Helicobacter pylori* is a common infection of the gastric mucosa that occurs early in childhood and tends to vary with age.

The prevalence of *H. pylori* infection among children with sickle cell anemia attending Mulago Hospital was 49%. This is a high prevalence of *H. pylori* and is consistent with findings from the study on healthy children in the Mulago community by Hestvik et al and lower than the prevalence among sick children admitted at Mulago hospital by Mugaba et al^18^, ^22^. Both of these local studies used the stool antigen test to determine *H. pylori* infection, similar to what was used in the current study.

The study by Mugaba et al which reported higher rates of 64% in a similar setting as ours was not limited to sickle cell anemia^18^. The possible explanation could be that sick children with SCA have different clinical and medication characteristics from other sick children in Uganda. Most of the children with SCA in Uganda had been on penicillin prophylaxis before their fifth birthday and some had pneumococcal vaccines and other unique medications which contribute to a low colonisation rate of the bacteria.

Karimi et al (2008) reported higher rates in Iran where the prevalence was 55% among children with sickle cell disease and recurrent abdominal pain^9^. A higher prevalence was found in the study by Sembanjo et al in Nigeria. In this study a high prevalence of 67.8% was reported^4^. We employed the stool test for an active *H. pylori* infection in our study compared to the serum ELISA based antibody test used by Sembanjo et al. This could have contributed to the differences in the prevalence rates between the two studies.

An Egyptian and Iranian study also reported higher rates of 79.2% and 68 % among adult patients with sickle cell disease and beta thalassemia, respectively^8^,^23^. Beta thalassemia is also a hemoglobin disorder with similar clinical characteristics to sickle cell disease. A difference in age categories between these two studies and the current study could explain the differences in prevalence.

Regarding the factors associated with *H. pylori* infection, having had epigastric pain at presentation was found to be independently associated with *H. pylori* infection among children with sickle cell anemia at Mulago Hospital. This was not surprising since epigastric pain has previously been reported as a common dyspeptic symptom also associated with *H. pylori* in the general population. This may also mean that children with sickle cell anemia are more likely to be symptomatic from *H. pylori* infection, since no local study has reported such an association previously. The protective factors against *H. pylori* infection included; having received pneumococcal vaccination and or having lost appetite for food at the time of presentation to the clinic.

The negative association between appetite loss and *H. pylori* infection is contrary to what has been found previously. *H. pylori* has been known to reduce appetite due to its effect on the gastric mucosa causing atrophic gastritis and downregulating the hormones Ghrelin and leptin which stimulate appetite^24^. The protective role of appetite loss may however be contributed to by the several possible causes of appetite loss in these children, like being on several medicines which may also reduce *H. pylori* infection, and other factors which were not studied in details. In addition, certain foods and nutrients like fruits and vegetables have been found to be effective for the prevention and eradication therapy of *H. pylori*, partly because of their antioxidant effect^25^. These foods are commonly used for children who have lost appetite to help boost their appetites. This could be of great public health importance to promote healthy diets. The dietary habits of our study participants were however not explored in details.

The protective role of pneumococcal vaccine may need to be studied further as limited data is available on the protective role of vaccines against *H. pylori* 26. This association can however be looked at in relationship with the socioeconomic status of the study participants as described below.

We did not find any positive association between low socioeconomic statuses that featured prominently in the study by Sembanjo et al^4^. This could have been contributed to by the relatively high education levels of caretakers in the current study, majority of whom had obtained secondary education and probably in fairer socioeconomic state. Temporal wasting which was a marker of undernutrition, commonly associated with low socioeconomic status was not associated with *H. pylori* infection in the current study. In another study done on children in Kampala by Hestvik et al, low socioeconomic status of the family was not associated with *H. pylori* infection^22^. However, the protective role of pneumococcal vaccination may be explained by the fact that those who can afford the vaccination are in a relatively better socioeconomic status, which may indirectly implicate low socioeconomic status in association with H. pylori infection.

Similar to what has been reported previously, there was no significant difference in gender association with *H. pylori* among the study participants in the current study. However age association was not in tandem with findings in other studies that found a decline in prevalence after 10 years of age attributed to spontaneous elimination of *H. pylori* infection^4^. There was no difference in the prevalence of *H. pylori* infection among those less than 12 years and those aged 12 years and above. We did not include those below 5 years of age for the challenges relating to the patient description of abdominal pain.

Recurrent abdominal pain had no independent association with *H. pylori* infection just as reported by Sembanjo et al in 2010. In contrast, Karimi et al 2008 found 54% of recurrent abdominal pain in sickle cell anemia patients attributable to *H. pylori* infection^9^, and Taghriid et al in 2011 reported significant positive association between *H. pylori* infection and recurrent abdominal pain among SCD patients^8^. A similar description of recurrent abdominal pain was used in these studies but ours was a larger, epidemiological study.

## Study limitations and strengths

We were unable to perform endoscopy with biopsy and histology of gastric specimen which is the gold standard test for presence of *H. pylori* infection. However, the stool antigen test used is highly sensitive at 95.6% and highly specific at 92.5% and has been used in other similar studies.

This is among the few studies that have studied *H. pylori* infection among sickle cell disease patients in Uganda. The study also evaluated one of the commonest /major symptoms of sickle cell anemia which is abdominal pain. We used a highly sensitive (95.6%) and highly specific (92.5%) *H. pylori* stool antigen test to detect active infection among our patients^21^. This implies a true reflection of the infection status compared to previous studies that may have exaggerated the magnitude of the infection based on the high sensitivity and poor specificity of the tests used.

## Conclusion

The prevalence of *H. pylori* among children aged 5–18 years with sickle cell anemia attending Mulago Hospital is high at 49% and epigastric pain was strongly associated with *H. pylori* infection among these children. However; recurrent abdominal pain was not. Pneumococcal vaccination and appetite loss at presentation were protective against *H. pylori* infection.

## Recommendations

Children with sickle cell anemia presenting with dyspeptic symptoms particularly epigastric pain should be routinely assessed for presence of *H. pylori*.

A longitudinal or cohort study should be done to further establish the role of pneumococcal vaccination in protecting against *H. pylori* infection among SCA patients.

## Data Availability

The datasets used and/or analysed during the current study are available from the corresponding author on reasonable request.

## List of abbreviations

aOR: Adjusted odds ratio
BMI: Body Mass Index
CI: Confidence Intervals
ELISA: Enzyme-linked Immunosorbent assay
HbS: Sickle cell hemoglobin
HbSS: Homozygous Sickle Cell Disease
H. pylori: Helicobacter pylori
NSAIDs: Non-steroidal anti-inflammatory drugs
RAP: Recurrent Abdominal Pain
SCA: Sickle Cell Anemia
SCD: Sickle Cell Disease
SD: Standard deviation
WHO: World Health Organization

## Authors' information

ISM: MBChB MMED (Mak). Was a resident (Student of Masters of Medicine Paediatrics and Child health) at Makerere University College of Health Sciences, by the time of the study, but now a Paediatrician at Kawempe National Referral Hospital for the last 27 months.

PGK: MBChB, MMED (Mak), Paediatrician Mulago National Referral Hospital.

HH: MD, FRCPC, Dept of Paediatrics, CHUSJ, Universite de Montreal, Montreal, QC, Canada.)

GN: MBChB, MMED (Mak), PhD, Professor of Paediatrics Makerere University College of Health Sciences
